# Integrating mental health care to reduce intimate partner violence in complex humanitarian emergencies

**DOI:** 10.1016/S2214-109X(25)00191-3

**Published:** 2025-08

**Authors:** Christine Bourey, Sarah M Murray, Wietse A Tol, Judith K Bass, Aissata Ba, Bathsheba Mahenge, Saphira Mulemba, Kathryn Falb

**Affiliations:** **Department of Epidemiology, Columbia University Mailman School of Public Health, New York, NY, USA** (C Bourey PhD)**; Department of Mental Health, Johns Hopkins Bloomberg School of Public Health, Baltimore, MD, USA** (S M Murray PhD, Prof J K Bass PhD)**; Section of Global Health, Department of Public Health, University of Copenhagen, Copenhagen, Denmark** (Prof W A Tol PhD)**; Athena Institute, Vrije Universiteit Amsterdam, Amsterdam, Netherlands** (Prof W A Tol PhD)**; University of Sciences, Techniques, and Technologies of Bamako, Bamako, Mali** (A Ba MA)**; School of Medicine and Dentistry, University of Dodoma, Dodoma, Tanzania** (B Mahenge PhD)**; Department of Mental Health, Centre for Infectious Disease Research in Zambia, Lusaka, Zambia** (S Mulemba MPH)**; Department of International Health, Johns Hopkins Bloomberg School of Public Health, Baltimore, MD, USA** (K Falb ScD)

## Abstract

The public health burden of intimate partner violence (IPV) is immense, particularly in complex humanitarian emergencies, where up to three in four women report experiencing lifetime IPV. Informed by feminist theory, current interventions addressing IPV in these settings often use gender-transformative approaches to advance more equitable gender attitudes, community mobilisation efforts to engage men in changing gender norms, and economic-focused programming to advance equitable financial decision making within couples. In this Viewpoint, we argue that feminist-grounded efforts to reduce IPV might benefit from incorporating interventions specifically targeted towards improving mental health. Taking settings affected by armed conflict as an example, we reflect on the utility of integrating mental health interventions into IPV programming and highlight three innovations and approaches to advance these efforts. Fundamentally, we aim to support researchers’ and interventionists’ incorporation of mental health care into gender-transformative programming in a robust manner, to reduce the burden of IPV in complex humanitarian emergencies.

## Introduction

Although the UN included eliminating violence against women in the Sustainable Development Goals,^1^ it remains a widespread public health issue. Intimate partner violence (IPV) is particularly prevalent, affecting more than one in four women globally, and has severe consequences for physical, mental, and reproductive health.^2^ In response, IPV researchers and practitioners have developed effective interventions that, particularly in low-income and middle-income countries (LMICs), include gender-transformative approaches that target patriarchal attitudes and norms, which are a root cause of violence against women.^3^ Despite these advances in reducing IPV, many communities affected by complex humanitarian emergencies remain without effective, sustainable, and scalable IPV interventions. To address this formidable challenge, we present recommendations and avenues for integrating mental health care to bolster the effect of IPV programming in humanitarian settings. As in the *Lancet Psychiatry* Commission on IPV and mental health,^4^ we do not intend for the focus on mental health to exclude key existing approaches. Interventions should continue to target the most salient drivers of violence in each setting,^5,6^ but can be enhanced through an integrated focus on mental health.

We focus on humanitarian settings affected by armed conflict, where research on IPV prevention and response has yielded mixed results. Conflict-affected settings are globally prevalent—2 billion people reside in conflict-affected, vulnerable, or fragile settings^7^—and they have the potential for a high prevalence of IPV.^8^ This high prevalence probably occurs due to complex processes involving impunity for, and normalisation of, violence, perceived challenges to masculinity, extreme poverty and related food insecurity, and potentially traumatic experiences resulting in a high burden of poor mental health, among other factors.^9^ Although patriarchal norms influence how many of these processes contribute to IPV, evidence supporting gender-transformative interventions to reduce IPV in conflict-affected settings is inconsistent. For example, of seven interventions that engaged men in challenging patriarchal norms, attitudes, and behaviours to reduce IPV in conflict-affected settings, only one led to reductions in IPV in the general population.^10^ Another reduced IPV only among men who were the most physically violent,^11^ and a third intervention showed reductions only among couples who most adhered to the intervention.^12^ Gender-transformative interventions that engaged only women have been rare; we identified only one, which showed a reduction in IPV.^13^ This mixed evidence might partly reflect methodological and operational challenges inherent in conflict-affected settings.^14^ Simultaneously, these results present an opportunity to examine why such interventions seem more broadly effective in stable settings than in conflict-affected settings, and to explore ways to enhance IPV interventions in these settings.

One contributing factor to these mixed findings might be the inadequate adaptation of evidence-based approaches to address the complex drivers of IPV in conflict-affected settings, such as poor mental health. One framework suggests that armed conflict worsens structural factors (gender inequality, normalised violence, and poverty) and individual or relationship factors (poor mental health, childhood neglect, and disability) that drive IPV perpetration (and victimisation);^15^ however, empirical evidence suggests that armed conflict affects these factors unevenly. For example, one analysis highlighted three pathways with evidence for increased IPV perpetration in conflict-affected settings: men’s gender-inequitable attitudes, exposure to interpersonal violence, and trauma symptoms.^16^ By contrast, conflict-related violence and socioeconomic status did not increase the odds of IPV perpetration when other factors were considered.^16^

Conflict-affected settings have a high burden of mental ill health, resulting from both direct exposure to conflict and family violence,^17^ and indirect effects such as poverty, displacement, disrupted social networks, and weakened civil society.^18^ The prevalence of post-traumatic stress disorder (PTSD) and depression are more than twice the global average,^19^ and alcohol use is also elevated in some conflict-affected settings.^20^ Although all mental health conditions can emerge or worsen in these contexts, we focus here on common mental disorders (eg, depression, anxiety, and PTSD) and substance misuse. Severe mental disorders (eg, bipolar disorder and schizophrenia) typically require clinical care rather than integration into gender-transformative interventions. We acknowledge that addressing gender equity and IPV is essential in clinical settings as well.^21,22^

## Mental health and IPV

The complex associations between mental ill health and IPV underscore the potential for mental health interventions to contribute important strategies to reduce IPV. For women, a bidirectional association exists that can result in cycles of victimisation and poor mental health. Experiencing IPV represents a potentially traumatic event with mental health consequences, and these consequences can mediate cycles of victimisation and revictimisation. Similarly, for men, associations between PTSD, depression, anxiety, and alcohol misuse and IPV perpetration have been documented.^23,24^ In relationships, women’s and men’s mental health are inextricably connected to one another and to relationship satisfaction and discord.

These interconnections suggest that mental health interventions could be a valuable component of IPV prevention and response. For women who have experienced IPV, reducing emotional numbing and hyperarousal (symptoms of PTSD) could help them better recognise and respond to potential future threats. Similarly, interventions that enhance hope and self-esteem—often diminished by depression—could strengthen survivors’ sense of agency and ability to keep themselves safe. Some men who perpetrate IPV might have deficits in information processing, such as misinterpreting a partner’s ambiguous behaviour as rejection, and could have reduced impulse control due to trauma exposure, making them prone to aggressive reactions, among other pathways.^25,26^ For both women and men, mental health interventions can also build psychological and interpersonal skills, such as stress management and communication, which reduce relationship conflict and decrease the risk of IPV.

## Current evidence

A Cochrane review of psychological therapies for women who have experienced IPV showed inconsistent evidence about the beneficial effect of psychological therapies in reducing re-exposure to IPV.^27^ However, three reviews of mental health interventions for IPV provide preliminary evidence that treatment for common mental disorders might reduce IPV in LMIC settings.^28–30^ Although the mental health interventions in these reviews were mostly implemented in stable LMIC settings, many of the studied communities have more in common with conflict-affected LMIC settings (eg, frequent economic deprivation and high burden of inequitable gender norms), than the USA, Canada, and western Europe. Mental health interventions have reduced at least one form of IPV in China among adult women and men with depression or PTSD (Interpersonal Psychotherapy);^31,32^ in India among adult women and men with depression (Healthy Activity Program);^33^ in Rwanda among families affected by HIV (Family Strengthening Intervention);^34^ and in Zambia among adult women survivors of IPV and men with hazardous alcohol use (Common Elements Treatment Approach).^35^ Only a few studies have been done in conflict-affected settings, and the results have been mixed. A trial in eastern Democratic Republic of the Congo (Narrative Exposure Therapy for Forensic Offender Rehabilitation) offered narrative exposure therapy to men who were former combatants and child soldiers and found a reduction in current violent behaviour that included IPV.^36^ By contrast, a small feasibility trial of an integrated psychosocial intervention combining Cognitive Processing Therapy and advocacy counselling among female Congolese refugees found mixed and inconclusive results,^37^ and an underpowered trial of Interpersonal Psychotherapy for Sudanese refugees in Egypt found no effect on IPV.^38^

Despite a strong theoretical rationale, high burden of poor mental health, and emerging proof of concept from LMICs, evidence on the use of mental health interventions to prevent or reduce IPV in conflict-affected settings remains scarce. Rigorous research is clearly needed to examine if and how addressing mental health of both women and men in these impacted communities can prevent and reduce IPV. In this Viewpoint, we lay out recommendations for programmatic and research priorities that leverage mental health interventions.

First, develop and evaluate integrated approaches that combine gender-transformative and mental health strategies in a complementary, synergistic manner.

Second, generate evidence on the mechanisms through which mental health interventions affect IPV outcomes, with the goal of streamlining interventions and supporting scalability.

Finally, position mental health as one component of a broader, inclusive, skills-based, and strength-oriented framework that addresses the structural and relational drivers of IPV in humanitarian contexts.

## Recommendations

### Build synergistic mental health care and multilevel feminist interventions

Effective intervention design requires a robust theory of change, attention to multiple drivers of IPV, integrated support for survivors, and engagement with both women and men. Integrated mental health care and gender-transformative interventions align with these principles, addressing multiple IPV risk pathways across socioecological levels. Although gender-transformative interventions can target structural, community, and interpersonal gender inequities, mental health interventions, including psychotherapies for symptomatic mental disorders, can influence intrapersonal and interpersonal dynamics linked to IPV. These interventions could play a key role in helping men engage with the social norm changes promoted by gender-transformative interventions. We hypothesise that increasing men’s mindfulness, psychological flexibility, and capacity for cognitive reappraisal through mental health treatment can increase their ability to learn and drive change in social norms and IPV. This idea builds on the concept of mental bandwidth, which describes the ability to perform basic functions that support higher-order behaviour and decision making.^39^ Although poverty and increased mental health symptoms can reduce mental bandwidth, psychotherapy can possibly improve decision making by helping individuals to better allocate their mental resources.^40^ Additionally, mental health interventions could enhance psychological flexibility, which is the ability to choose prosocial and values-based behaviour even in the presence of psychological barriers such as anger or shame. Past research suggests that psychotherapies that enhance psychological flexibility and encourage values-driven action, such as Acceptance and Commitment Therapy, can support non-violent behaviours in men who perpetrate IPV.^41^ For women, combining mental health care and multilevel feminist interventions might enhance self-esteem and self-efficacy, helping them to address and resolve conflict and to assert their needs and boundaries when safe to do so. Thus, mental health care could be an underexplored yet crucial component of strengthening relationships, as outlined in the evidence-based RESPECT Framework.^42^

### Identify the most relevant mental health intervention components and their mechanisms

A range of interventions—from psychosocial support programmes that include mental health components (eg, emotion regulation skills) to structured psychotherapies—should be tested alongside gender-transformative programming. For example, for women, interventions could include brief skills sessions aimed at enhancing stress management and self-efficacy, as well as more intensive approaches such as Cognitive Processing Therapy to address trauma-related cognitive patterns. Existing compilations of components supported by evidence for reducing mental health symptoms among women survivors of IPV can serve as a useful starting point for adaptation and testing.^43^

The specific mental health components or interventions to include, and at what intensity and duration, will vary based on population and setting. Given the current evidence base, making firm recommendations remains challenging. As combinations of services are piloted and evaluated, evidence needs to be generated on the psychological mechanisms linking poor mental health to IPV experience and perpetration. Identifying these mechanisms can guide the selection of targeted, scalable components to be incorporated into broader gender-transformative programmes.

We emphasise psychosocial and psychotherapeutic interventions rather than medications because evidence is sparse for the efficacy of medication in reducing IPV. There also needs to be better availability of WHO essential medicines in humanitarian settings.^44,45^ Furthermore, effective models for medication prescription often depend on integrating mental health into primary care services.^46,47^

### Focus on inclusive, holistic intervention

Mental health interventions should be integrated into holistic, skills-building, strength-based approaches to addressing IPV. We contend that mental health interventions should not be designed solely to engage people who meet criteria for specific mental disorders. Focusing exclusively on those with high symptoms could support a false narrative that people living with mental disorders are dangerous, thereby generating or increasing stigma associated with, and potentially discouraging participation in, interventions or causing social harm. Psychosocial programming and psychological care that promote mental wellbeing, prevent mental ill health, and reduce the burden of mental ill health often associated with living in a conflict-affected setting are social goods to which everyone should have access, although, in reality, few do. Within this framework, supporting women who are currently experiencing IPV should be a priority, including addressing their mental health needs.^48^ Engagement with men who perpetrate violence with mental health interventions needs to be approached with care, ensuring that the environment remains safe, healing, and trusting for survivors.

## Conclusion

Addressing poor mental health might help to reduce IPV in conflict-affected settings, where the burden of mental ill health is particularly high. We have discussed the potential benefits of mental health interventions for both women and men. In practice, these services are typically accessed by women, despite the prevalence of common mental disorders being similar for women and men in conflict-affected settings.^17^ Engaging with men will require specific attention to how gender norms influence recruitment, engagement, and retention in mental health services especially.

This Viewpoint focuses on conflict-affected settings because specific stressors are shared across these areas, and we are appropriately positioned to suggest improvements as humanitarian health researchers and practitioners. Providing mental health interventions could be a priority in other violence-affected settings where the burden of poor mental health is high, and care that targets trauma and substance misuse has proven more effective at reducing IPV perpetration among men than has alternatives. However, the integration of gender-transformative and mental health interventions might not be a priority in many violence-affected settings globally. Interventions should be tailored to the specific contexts and drivers of violence in each setting.

The imperative to incorporate mental health interventions into IPV programming should not be seen as placing blame on women who have experienced IPV or excusing the actions of men who perpetrate this violence, who might also have poor mental health. Rather, this approach recognises that exposure to violence affects both women and men and, in the context of unequal power dynamics, produces a modifiable mental health pathway that can be addressed to support everyone’s right to mental wellbeing and to mitigate the risk of IPV.

Efforts to adopt more holistic approaches should not come at the financial expense of survivor-centred responses for women, nor should they negatively affect women’s ability to seek healing and care. Donors should be pushed to support long-term, multipronged programming and learn to disentangle and disrupt the complex interactions between violence in the community, IPV, and mental health to improve the wellbeing of current and future generations.
